# Effect of positional dependence and alignment strategy on modeling transcription factor binding sites

**DOI:** 10.1186/1756-0500-5-340

**Published:** 2012-07-02

**Authors:** Saad Quader, Chun-Hsi Huang

**Affiliations:** 1 Department of Computer Science & Engineering, University of Connecticut, 371 Fairfield Road, Storrs, Unit 2155, Connecticut 06269-2155, USA

## Abstract

**Background:**

Many consensus-based and Position Weight Matrix-based methods for recognizing *transcription factor binding sites* (TFBS) are not well suited to the variability in the lengths of binding sites. Besides, many methods discard known binding sites while building the model. Moreover, the impact of *Information Content* (IC) and the positional dependence of nucleotides within an aligned set of TFBS has not been well researched for modeling variable-length binding sites. In this paper, we propose *ML-Consensus* (Mixed-Length Consensus): a consensus model for variable-length TFBS which does not exclude any reported binding sites.

**Methods:**

We consider *Pairwise Score* (PS) as a measure of positional dependence of nucleotides within an alignment of TFBS. We investigate how the prediction accuracy of ML-Consensus is affected by the incorporation of IC and PS with a particular binding site alignment strategy. We perform cross-validations for datasets of six species from the TRANSFAC public database, and analyze the results using ROC curves and the Wilcoxon matched-pair signed-ranks test.

**Results:**

We observe that the incorporation of IC and PS in ML-Consensus results in statistically significant improvement in the prediction accuracy of the model. Moreover, the existence of a core region among the known binding sites (of any length) is witnessed by the pairwise coexistence of nucleotides within the core length.

**Conclusions:**

These observations suggest the possibility of an efficient multiple sequence alignment algorithm for aligning TFBS, accommodating known binding sites of any length, for optimal (or near-optimal) TFBS prediction. However, designing such an algorithm is a matter of further investigation.

## Background

Transcription factors (TF) are proteins that bind to specific locations of DNA (referred to as *binding sites*, BS) and facilitate/repress the transcription process. In many cases binding sites of a transcription factor contain a common nucleotide pattern [1]. DNA motif-finding algorithms use various models to represent this pattern [1]. One of these models is the *consensus*, a sequence representation derived from a multiple sequence alignment of binding sites [2,3]. The consensus sequence retains only the most conserved base at any position, resulting in loss of information about other bases at that position. *Position weight matrix* (PWM), also known as *probabilistic sequence model* or *scoring matrix*, is another representation model which records frequency (or probability) of every base at each position of the multiple sequence alignment [1,4,5]. The survey by Das and Dai provides a classification of DNA motif-finding methods based on different representation models [6].

Both basic consensus-based and PWM-based methods need equal-length sequences. Although this is acceptable for cases where there is no variability in lengths of binding sites (e.g., the bacterial dataset described in [7]), there are other datasets where TFBS show remarkable variability in lengths (e.g., datasets described in Section Input, training, and testing). In order to circumvent this variability, variants of these methods apply constraints and assumptions on the nature of binding sites. For example, only fixed-length sites are considered, or only sites containing a fixed-length subsequence are considered [8]. It is not confirmed, however, whether the protein-DNA binding mechanism indeed follows such constraints. Therefore it is necessary to modify these models for allowing variability in TFBS lengths. Some studies described such a PWM-based model that allows gaps in the PWM and thus accommodates variable-length binding sites [9].

There are models which can accommodate binding sites of different lengths. A widely used TFBS prediction program is the PMATCH, which uses Gibbs sampling [10] to align binding sites of different lengths [11]. However, PMATCH excludes some documented binding sites based on constraints on the lengths of the sites, and imposes a constraint on the the core region; it defines the core region as the five most conserved positions within the alignment [11].

Models that involve a matrix representation (PWM/consensus) must make a multiple sequence alignment from the known binding sites. Therefore, the multiple sequence alignment algorithm associated with such a model will influence its performance because the alignment (and therefore, the scoring matrix or consensus) generated by different algorithms will be different. An excellent survey of multiple sequence alignment algorithms can be found in [12]. On the other hand, the TFBS prediction algorithm SiTaR does not align input sequences at all [13]. By not aligning, SiTaR avoids many uncertainties arising from the generalizations made by multiple sequence alignment.

Basic consensus-based and PWM-based models assume that positions in a binding site are independent. However, some biological studies suggest that positions in a binding site are correlated [14,15]. Several computational models for this correlation have been proposed [16,17]. Some studies described *pairwise score* (PS), a method that computes interdependence of any two positions that are located within a fixed distance from one another in a binding site [18]. This distance is called the *scope* of PS. It has been shown that the addition of PS to basic consensus-based and PWM-based models results in statistically significant improvement in performance [18]. However, pairwise correlation is not the same as the statistical measure “correlation”; rather, it is a measure of co-occurrence of bases within a given proximity (i.e., scope). The mathematical definition of pairwise score can be found in Section Scoring function with pairwise score (PS).

*Information content* (IC) of an alignment of binding sites is a measure of conservation of any base at any given position in that alignment. It has been shown that the addition of IC in basic consensus-based and PWM-based models results in statistically significant improvement in performance [18]. However, these results regarding PS and IC were demonstrated on a dataset that does not have any variability in the lengths of binding sites for a TF [7].

### Our research

In this paper, we define a consensus model (*Mixed-length Consensus* or *ML-Consensus*) for recognizing variable-length TFBS. Our model does not exclude any known/reported binding site while building the model for a set of TFBS. Moreover, ML-Consensus does not make any assumption on the lengths of binding sites or on the length/composition of the core region. However, it assumes that there exists one core region for a set of TFBS, the core region is present, in part or whole, in every binding site. This assumption is used in constructing the naïve multiple alignment algorithm associated with this model (described later in this section).

Our input data covers six species from the TRANSFAC public database [19]. We study the effect of pairwise correlation of nucleotides, information content, and multiple sequence alignment strategy on the prediction accuracy of our model.

If each binding site of any given TF has the same length (e.g., the *E. coli* dataset in [7]), it is trivial to align them and get the consensus or scoring matrix. Otherwise, one needs to make a multiple sequence alignment from those sequences in order to derive a scoring matrix or a consensus. TFBS prediction tools employ various methods for aligning binding sites [6]. All other things remaining the same, effectiveness of two multiple sequence alignment algorithms for aligning TFBS can be evaluated by comparing the performance of a TFBS prediction model using those two alignment strategies.

In our study, our goal was to evaluate the effectiveness of commonly used multiple sequence alignment strategies in aligning TFBS. We present a naïve sorting-based multiple sequence alignment algorithm and compare it to ClustalW2, a widely used multiple sequence alignment algorithm [20]. We pick ClustalW2 as a representative of sophisticated alignment algorithms; our simple-sorted alignment algorithm is so naïve that when comparing it to another algorithm the implementation specifics of the other algorithm does not matter provided the other algorithm is one of the good and sophisticated algorithms. Our algorithm (see Appendix A: The naïve sorting-based multiple sequence alignment algorithm) operates on a simple principle: it picks the shortest yet-to-align sequence and adds it to the temporary alignment. This is done based on the assumption that all binding sites of a TF have some pattern in common (i.e., a core region), and therefore the probability that any given position of a binding site would be a part of the core region is higher in a short binding site than that in a long binding site. On the other hand, ClustalW2 creates the alignment from the phylogenetic tree built from pairwise alignments from the input sequences. In our experiments, we used ClustalW2 without iterative refinement. Table 1 shows different alignments produced by these two algorithms from the same input sequences.

**Table 1:** Consensus derived from binding sites aligned using ClustalW2 (left) and simple sorted (right) multiple sequence alignment algorithms

**ClustalW2 Alignment**	**Simple Sorted Alignment**
-------ATTACACCAAGTACC	-------ACCTAAGCTG--
----GGAATTTCCTGTTGATCC	----ATTACACCAAGTACC
-------ACCTAA-GCTG----	-GGAATTTCCTGTTGATCC
CTAAAGGACGTCACATTGC---	CTAAAGGACGTCACATTGC
-------A--TCA---TG----	----A--AC-T-A--T--C

Pairwise score (PS) is a measure of the dependence of nucleotides at two positions that are situated within a given distance in an alignment of TFBS. Whereas other studies (e.g., [18]) have discussed effect of PS on consensus/matrix-based TFBS models for fixed-length binding sites, we study the effect of PS on ML-Consensus, a model for variable-length binding sites. Specifically, we perform experiments with different PS scopes to find whether there is any regularity with which a change in PS scope affects the performance of ML-Consensus. We consider the following choices for PS: no PS, PS scopes 1–10, and a large scope value that covers the entire overlap between a test sequence and the consensus while scoring that sequence against the consensus.

As mentioned earlier, ML-Consensus has three configuration variables: pairwise score, information content, and multiple sequence alignment strategy. We construct one experiment-configuration for each combination of variables (e.g., ClustalW2 alignment using IC and PS scope 2, etc.). We conduct leave-one-out cross-validation scheme for training/testing our model on TFBS data for six species extracted from TRANSFAC public database [19]. We used ROC curves and the Wilcoxon matched-pair signed-ranks test for statistical evaluation of the performance data.

Our results show that the adoption of IC or PS in the scoring function of ML-Consensus results in significant improvement in performance. Moreover, a large PS scope (e.g., the full scope) does not produce the best performance for a given configuration; performance decreases after PS scope is larger than a certain value. Not only is this observation counterintuitive, but it also provides a way to estimate the core length. Our results also suggest that it is possible to design a TFBS-specific multiple sequence alignment algorithm that will perform better than general-purpose algorithms by means of utilizing prior information and assumptions about TFBS. However, we do not present such an algorithm yet since it is subject to further investigation.

The main contributions of this paper are the following: (1) We describe a new model for TFBS prediction which accommodates all known binding sites of different lengths. (2) We show that incorporating information content and pairwise correlation into scoring function for this model improves the prediction accuracy. (3) We study the effect of different PS scopes on the prediction accuracy of this model. (4) We show that it is possible to estimate the length of the core region in a set of TFBS, and (5) We show that it is possible to design a multiple sequence alignment algorithm which will do better than general-purpose algorithms while aligning TFBS.

## Results and discussion

In the following discussion *AUC* refers to the area under ROC curve. A *configuration* is an experiment with any particular settings for IC, PS scope, and alignment strategy. AUC of a configuration is taken as a measure of its performance (i.e., prediction accuracy). However, when comparing performances of two configurations, the statistical significance of difference in performance is considered. If significant, the event is mentioned as *configuration A performs better than configuration B*. Otherwise, it is mentioned as *the two configurations are equivalent*. For a given configuration, *peak in its AUC* denotes the PS scope value which, among all scopes, produces the highest AUC for that configuration. The phrases *naïve alignment*, *simple sorting-based alignment* and *simple-sorted alignment* all refer to our heuristic, sorting-based, multiple sequence alignment algorithm presented in Appendix A: The naïve sorting-based multiple sequence alignment algorithm.

### Some adjacent PS scopes produce significantly better performance than other scopes

**Figure 1:**
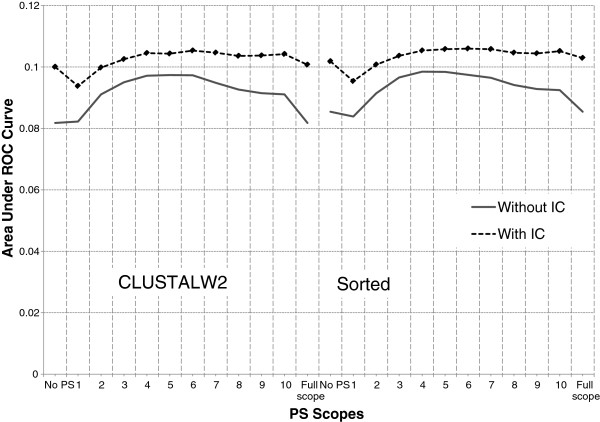
**Effect of IC on alignment and PS ( *H. sapiens* ).** There are two regions: ClustalW2 (left) and simple sorting-based algorithm (right). In each region, PS scopes are placed in x-axis, from left to right, in the following order: no PS, scopes 1–10, and full scope. Between the two configurations (one using IC, and the other without IC), if one performs significantly better than the other at any PS scope, it is marked with a diamond.

**Figure 2:**
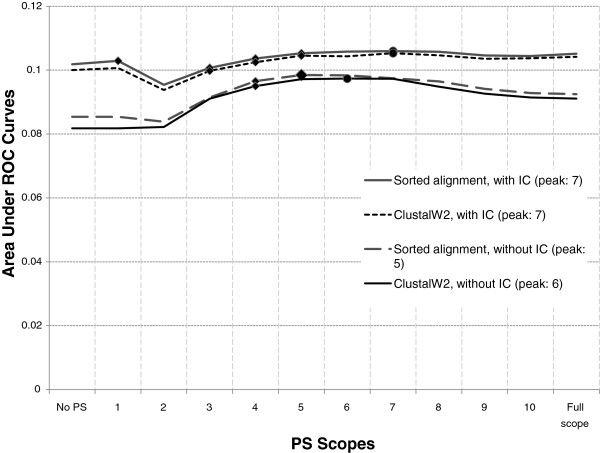
**Performance of all configurations in *H. sapiens* .** If a PS scope produces significantly better performance than that produced by its previous scope *and* is followed by a scope where the performance does not change significantly, it is marked with a diamond (the start of the significance plateau). After this point, if the performance does not change significantly in subsequent scopes, data points are still marked with a diamond (continuation of the plateau). A round marker denotes the highest AUC in a configuration. A large diamond indicates that the scope producing this peak in AUC is inside the significance plateau.

By definition (see Section Scoring function with pairwise score (PS)), all information gathered in a smaller PS scope are retained in a larger PS scope. However, Figure 1 and Figure 2 show that the performance of a configuration starts to decrease when PS scope grows larger than a certain value. The location of the peak (i.e., the PS scope which produces the highest area under ROC curve) for a configuration varies in different species. Figure 2 depicts whether the change in performance between successive PS scopes is statistically significant. We observe that there is always a range of PS scopes where performance, after initially increasing significantly, does not change significantly with a change in PS scope. After this range, however, performance decreases significantly. We call this range of PS scopes a *significance plateau*.

The above observation can be explained as follows. For any leave-one-out experiment over a given set of TFBS, the known positive example may have one or more mismatches with respect to the consensus. These positions may get involved in position-pair matches between the consensus and a known negative example. (We term this event as *noise*.) If such an event takes place, it increases the probability that the known negative example would score higher than the known positive example — producing a false positive. PS scopes larger than a certain value do not capture any new position-pair matches, yet continue picking up noise. This is why we observe a decrease in performance of a configuration with increase in PS scopes beyond a certain value. This scope indicates the maximum distance within which two positions in an aligned set of TFBS are correlated. In an alignment, only positions that form the core region will be correlated. Therefore the core region (for the sites in the alignment) should be at most as long as this scope value. However, this scope value is found by running a given experiment-configuration over all sets of TFBS for a given species, and therefore it is associated with the overall TFBS dataset for the species and not with any particular set of TFBS. Additionally, different experiment-configurations produce possibly different significance plateaus for any given species dataset. Therefore, the location of the plateau depends on which experiment-configuration is in use. Our suggestion is that for a given species, we should choose the experiment-configuration that produces highest area under its ROC curves across all PS scopes, thus having the highest discriminatory power.

### ClustalW2 does not perform as expected

We perform the Wilcoxon matched-pair signed-ranks test in order to determine whether ClustalW2 performs significantly better than the naïve alignment algorithm. Since ClustalW2 is a sophisticated algorithm, the null hypothesis is that ClustalW2 should perform *significantly better* (with *p*<=0.05) than simple sorted alignment algorithm in *all* combinations of other variables. However, if the difference in performance is found insignificant it should be considered as an evidence against the null hypothesis. We divide all configurations into pairs (based on alignment strategy), and then compute statistical significance of difference in performance of the two configurations in each pair. Table 2 shows the statistical significance of the difference in performance of configurations using different alignment algorithms according to the null hypothesis mentioned above. It can be seen that the null hypothesis does not hold true in four out of six species with *p*<=0.01. This means ClustalW2 does not perform significantly better than naïve sorting-based alignment strategy in all experiments.

**Table 2:** Comparison between ClustalW2 and simple sorted alignment strategies in all configurations

**Species**	**ClustalW2**	**Superior**	** *Z* **	** *p≤* **
	** % Cases**	**Alignment**		
*D. melanogaster*	0.980	ClustalW2	5.78	0.01
*G. gallus*	0.000	Simple sorted	6.03	0.01
*H. sapiens*	0.540	None	0.50	–
*M. musculus*	0.060	Simple sorted	5.28	0.01
*R. norvegicus*	1.000	ClustalW2	6.03	0.01
*S. cerevisiae*	0.125	Simple sorted	4.53	0.01

Since the naïve algorithm operates on simple assumptions, and it does not do anything as involved as common multiple sequence alignment algorithms do, the naïve algorithm has much room for improvement. Since the performance of this algorithm is already as good as (or better than) the performance of ClustalW2 in most situations, we can say that it is possible to design a TFBS-specific multiple sequence alignment algorithm that will perform better than general-purpose algorithms (e.g., ClustalW2, etc.) by means of utilizing prior information and assumptions about TFBS. For example, an assumption made by the naïve alignment algorithm is that there is a core region contained by all binding sites. In addition, an example of prior information about TFBS is the core length suggested by the PS scopes in significance plateau. However, the relationship between the core length and the significance plateau is not known yet.

**Figure 3:**
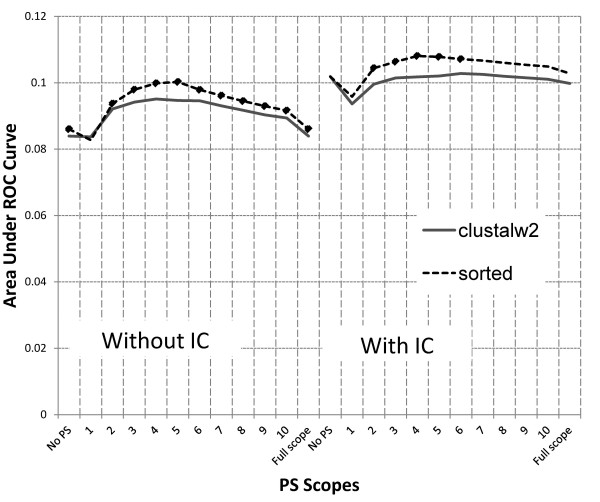
**Effect of alignment on IC and PS ( *M. musculus* ).** A diamond marker indicates where a configuration performs significantly better than the other in the same PS scope.

**Figure 4:**
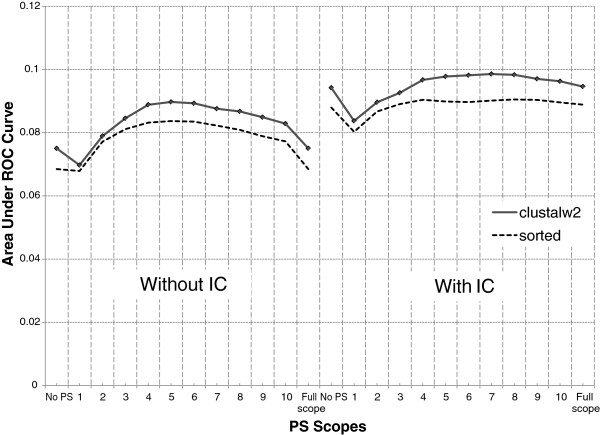
**Effect of alignment on IC and PS ( *R. norvegicus* ).** A diamond marker indicates where a configuration performs significantly better than the other in the same PS scope.

Performance of configurations using the same alignment algorithm varies across different species. Figure 3 shows that simple sorted alignment performs better than ClustalW2 in *M. musculus*. On the other hand, Figure 4 shows that ClustalW2 performs better than naïve sorting-based algorithm in *R. norvegicus*. Although we do not know why this happens, our hypothesis is that it may be due to the differences in the composition of binding sites (i.e., number and lengths of binding sites, the nature of the core region, etc.) for each species. This observation requires further investigation.

### Both IC and PS lead to improved performance

The addition of IC to a configuration without IC always improves its performance. However, the improvement is more prominent for larger scopes which can be seen in Figure 1 and Figure 2. In these figures, AUC of configurations without IC drops quickly at large PS scopes. However, curves for configurations with IC tend to be more flat at large PS scopes. Similarly, addition of PS (with appropriate scope value) to configurations without PS results in improved performance. These observations are in accordance with the observations made by [18] regarding influence of IC and PS on models for fixed-length binding sites.

## Conclusions and future works

In this paper we describe ML-Consensus, a consensus model for recognizing variable-length transcription factor binding sites. We show that certain PS scope values indicate the range within which positions in a binding site are correlated. However, the statistical correlation of nucleotides in a set of binding sites is out of the scope of this research, and is a matter of future work. We also show that in most cases, configurations that use ClustalW2 as alignment algorithm do not perform significantly better than configurations that use a naïve sorting-based heuristic alignment algorithm. It suggests that it is possible to improve the naïve algorithm into a TFBS-specific multiple sequence alignment algorithm (using information/assumptions about TFBS) which would perform better than general-purpose multiple sequence alignment algorithms. However, designing such an algorithm is another direction of future investigation. Lastly, although we use a consensus model, our approach and methods can be extended to a PWM-based model for variable-length binding sites.

## Methods

In this section, we start with presenting the mathematical definition of the ML-Consensus model and its various parts. Next we describe how we collected and processed the input data to build training and testing datasets. Then we describe how we made statistical evaluation of the experiments through ROC curves and Wilcoxon matched-pair signed-ranks test.

### Model definition

The ML-Consensus model has the following parts: (1) Building a multiple sequence alignment from a given set of binding sites, (2) Generating the consensus sequence from this alignment, (3) A basic scoring function which compares a given DNA sequence with this consensus and tells how close they are; this scoring function can be modified to incorporate information content (IC) and pairwise score (PS).

#### Building a consensus

Let *S* be the set of *N* binding sites for a particular transcription factor. Let *A* be a multiple sequence alignment of *S* with width of *M*. A gap in alignments in *A* is denoted by ‘-’.

Let *n*_*j*_(*b*) be the number of times base *b*∈{A,*C*,*G*,*T*} appears at *j*-th position of *A*. Let *f*_*j*_(*b*)=*n*_*j*_(*b*)/*N* be the corresponding frequency. Similarly, let *n*(*b*) be the number of times base *b* appears overall in *A*, and *f*(*b*) be the overall frequency for base *b* in *A*.

A letter representing more than one nucleotides is called the *ambiguity code* for those nucleotides. Let *amb*(*b*,*d*) be the ambiguity code for two bases *b*,*d*∈{A,*C*,*G*,*T*} as described in Table 3, and *amb*(*b*,∗) be any ambiguity code involving base *b*. Let *C* be the consensus sequence derived from *A*, and *C*_*j*_ be the *j*-th base in *C*.

**Table 3:** Ambiguity codes used in consensus for a pair of nucleotides

** *b* **	**A**	**A**	**A**	**C**	**C**	**G**
** *d* **	**C**	**G**	**T**	**G**	**T**	**T**
*amb*(*b*,*d*)	I	J	K	L	M	N

*C*_*j*_is computed as follows. For each position *j* of *A*, 

· If fj(b)>0.5 for base b∈{A,C,G,T}, set Cj=b.

· Otherwise, if fj(b) + fj(d)>0.75 for any two bases b,d∈{A,C,G,T}, set Cj=amb(b,d).

· Otherwise, set Cj= ‘-’, the gap character.

Table 1 shows how to derive a consensus from two different sequence alignments produced by two different alignment algorithms. Computing _*fj*_(*b*) for all *j*,*b*takes *O*(*NM*) time.

#### Scoring function

Let *t* be a putative binding site. Let _*tj*_be the *j*-th base of *t*. To compute the score of *t* with respect to consensus *C*, we used a sliding window approach where *t* is shifted along *C*, from left to right. At each point of shifting there is an overlap between *t* and *C*. For each overlap *w* let _*Cw*,*i*_be the base in consensus corresponding to the *i*-th position in *w*. Define _*tw*,*i*_ in similar way. For each overlap *w* we computed *σ*(*t*,*C*,*w*), the score of *t* at that particular overlap; this score is equal to the number of matches between *t* and *C* at *w*:

(1) $$\sigma (t,C,w)=\underset{i\in w}{\sum }\mathrm{Match}(w,i)\;,$$

where

(2) $$\mathrm{Match}(w,i)=\left\{\begin{array}{cc} 1 & :C_{w,i}=t_{w,i}\\ 1 & :C_{w,i}=\mathrm{amb}(t_{w,i},*)\\ 0 & :\mathrm{otherwise} \end{array}\right)\;.$$

Computing *Match*(*w*,*i*) takes *O*(1) time, and computing *σ*(*t*,*C*,*w*) takes *O*(*M*) since size of *w* is *O*(*M*). Finally, the score of *t* with respect to *C* is the maximum score obtained in all overlaps, which takes *O*(*M*^2^) since there can be at most *O*(*M*) overlaps.

(3) $$\sigma (t,C)=\underset{w}{\max}\left(\sigma \left(t,C,w\right)\right)\;.$$

#### Scoring function with information content (IC)

Information Content (also called *entropy*) at any position *j* of the alignment *A* is a measure of conservation of any base at that position [4,21]. If a base is highly conserved at a position, chance of encountering a different base at that position is small; thus the information content at that position is low. The IC at position *j* of the alignment matrix *A* is defined as:

(4) $$\mathrm{IC}(A,j)=2+\underset{b\in \{A,C,G,T\}}{\sum }f_{j}(b)\log f_{j}(b)\;,$$

where the term $f_{j}(b)\log f_{j}(b)$ becomes zero whenever _*fj*_(*b*) becomes zero, thus avoiding evaluation of log0. *IC*(*Aj*) for all *j* can be computed in *O*(*M*) time. Let *A*(*wi*) be the position in *A* that corresponds to the *i*-th position in *w*. When IC is used in scoring, the scoring function for the overlap becomes:

(5) $$\sigma_{\mathrm{IC}}(t,C,w)=\underset{i\in w}{\sum }\mathrm{Match}(w,i)\cdot \mathrm{IC}\left(A,A\left(w,i\right)\right)\;.$$

This takes *O*(*M*) time when *IC*(*Aj*) are pre-computed.

#### Scoring function with pairwise score (PS)

Pairwise score is a measure of interdependence among positions in a binding site with respect to the consensus [18]. Two different positions in an overlap *w* are correlated if there are matches in both positions. In overlap *w*, let positions *i* and *i* + *k* be separated by *k* positions. The match-score for this position-pair, *MatchPair*(*wik*), is defined as follows:

(6) $$\mathrm{MatchPair}(w,i,k)=\left\{\begin{array}{cc} 2 & :\mathrm{Match}(w,i)=1\\ \text{and}\\ \mathrm{Match}(w,i+k)=1\\ 0 & :\mathrm{otherwise} \end{array}\right)$$

This takes *O*(1) time.

Let *K* be the maximum distance considered between any two positions, and |*w*| be the length of the overlap. *K* is called the scope of PS. The pairwise score of *t* at overlap *w*, _*σ*PS_(*t*,*C*,*w*), is defined as the total number of position-pair matches for all positions situated within the scope of PS.

(7) $$\sigma_{\mathrm{PS}}(t,C,w)=\overset{K}{\underset{s=1}{\sum }}\overset{|w|-s}{\underset{i=1}{\sum }}\overset{s}{\underset{k=1}{\sum }}\mathrm{MatchPair}(w,i,k)$$

This operation takes *O*(*MK*^2^) time. Score at any PS scope contains all matches from all smaller scopes along with new matches at the said scope. Thus it does not lose any information about position-matches gathered in previous scopes.

#### Scoring function with both information content and pairwise score

At any overlap *w*, let _*nij*_(*b*,*d*) be the number of times two bases *b* and *d* appear together at positions *i* and *j*, respectively. Let _*fij*_(*b*,*d*)=_*nij*_(*b*,*d*)/*N* be the corresponding frequency. Then, IC of position-pair (*i*,*j*) in the alignment matrix *A* is defined as follows:

(8) $$IC_{\mathrm{pair}}(A,i,j)=4+\underset{b,d\in \{A,C,G,T\}}{\sum }f_{\mathrm{ij}}(b,d)\log f_{\mathrm{ij}}(b,d)$$

Computing _*fij*_(*b*,*d*) for all *i*,*j*,*b*,*d* takes *O*(*M*^2^) time. After that, computing *I*_*C*pair_(*A*,*i*,*j*) for all *i*,*j*takes *O*(*M*^2^) time.

Let *A*(*w*,*i*) be the position in *A* that corresponds to the *i*-th position in *w*. Let *I*_*C*pair_(*w*,*i*,*k*) be the information content of the position-pair (*i*,*i* + *k*) in *w*, which is defined as follows:

(9) $$IC_{\mathrm{pair}}(w,i,k)=IC_{\mathrm{pair}}\left(A,A\left(w,i\right),A\left(w,i+k\right)\right)$$

Finally, the score of *t* at overlap *w* is defined as follows:

(10) $$\begin{array}{lll} \sigma_{\mathrm{ICPS}}(t,C,w)= & \overset{K}{\underset{s=1}{\sum }}\overset{|w|-s}{\underset{i=1}{\sum }}\overset{s}{\underset{k=1}{\sum }}\mathrm{MatchPair}(w,i,k)\; & \;\\ & \times \cdot \;\;IC_{\mathrm{pair}}(w,i,k)\;.\; \end{array}$$

This takes *O*(*K*^2^*M*) time because all *I*_*C*pair_(*A*,*i*,*j*) values are already computed for all *i*,*j*.

### Experiment design

We studied the effect of three variables on the performance of ML-Consensus: multiple sequence alignment strategy, IC, and PS. The alignment algorithm can be either ClustalW2 or simple sorted alignment algorithm. There are two choices for IC: either using IC, or not using IC. However, PS can have twelve possible values: not using PS; PS scopes 1–10; and lastly full PS scope, which means the scope spans the entire overlap between a putative site and the consensus. Therefore, there are 2×2×12=48 possible experiment-configurations, one for each combination of the three variables. Each of these configurations was trained and tested using the same input, training, and testing data.

### Input, training, and testing

**Figure 5:**
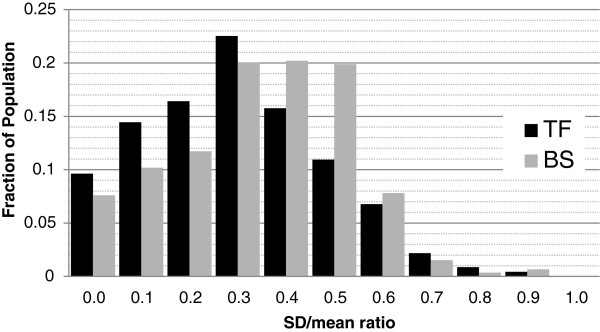
**Input variability.** Histogram for variability in TFBS length in our input data from Transfac Public database. Population standard deviation and mean have been counted for each set of TFBS. The x-axis shows the ratio of SD and mean which can be seen as a measure of variability in data. Each TF and all its BSs are assigned into one bin in x-axis. The y-axis shows the fraction of total TF or BS population that fell into each bin in x-axis.

We extracted TFBS data from TRANSFAC public database [19]. We considered TFs with at least three binding sites. Table 4 shows basic statistics for this data. Figure 5 shows the variability in TFBS lengths in the input data. The x-axis shows the ratio of population SD and mean in BS length computed for a set of TFBS. From the figure it can be observed that 9.5% TFs have small deviation in size (the first bin of histogram) but they cover only 7.5% of total BSs. From first three bins, it can be seen that 40% of TFs (covering 29% BSs) have low variability ($\frac{\mathrm{SD}}{\mathrm{mean}}<0.3$). From next three bins, it can be observed that another 49% TFs (covering 60% BSs) have much higher variability ($0.3\leq \frac{\mathrm{SD}}{\mathrm{mean}}<0.6$). Remaining 11% TFs have extreme variability, and they cover the remaining 11% of BSs.

**Table 4:** Statistics of input TFBS data

**Species**	**TF**	**BS**	**Average**	**Standard**
			**BS length**	**Deviation**
*D. melanogaster*	29	352	12.14	5.83
*G. gallus*	23	179	7.78	5.47
*H. sapiens*	179	2493	13.93	7.11
*M. musculus*	125	1266	10.13	6.01
*R. norvegicus*	59	795	13.47	6.80
*S. cerevisiae*	42	385	9.17	5.18
All species	457	5470	11.97	6.43

The Standard Deviation (SD) column is the average of population SD in BS lengths for all TFs.

We conducted leave-one-out cross-validation for all TFs over respective BS data. For each TF, the training data contained all its binding sites except the one left out. The test data contained all known negative examples (binding sites of other TFs of the same species) and one known positive example (the left-out site). In accordance with [18], we removed any site from the set of negative examples for this TF if the site is also a BS of this TF.

### Statistical evaluation

If a known negative example scored higher than the only known positive example, it was treated as a false positive. We needed to know which false positive rate corresponds to which true positive rate in order to draw an ROC curve for a configuration. In our case there was only one known positive example. Because several negative sites may score higher than the positive one, our model must allow these false positives (compromising prediction accuracy) in order to correctly classify the known positive example. We considered allowable false positive rate from 0% to 20%. This range was discretized into several slots (each denoting a smaller range in false positive rate values). For each leave-one-out experiment over a given set of TFBS we computed the true positive rate corresponding to each of these slots. These values were used to generate an ROC curve for this configuration. Details of this construction are given in Appendix B: Construction of ROC curves. Area under the ROC curve of a configuration is a measure of its discriminatory power. However, it should be noted that the performance of two configurations can not be compared solely by the areas under respective ROC curves if the two curves intersect at one or more points [22].

We used Wilcoxon matched-pair signed-ranks test to compare performance of any two configurations at *p*-values 0.05 and 0.01 [23]. This test is well suited to our experiments because the underlying distribution of the data is unknown, yet we know that individual data points are independent. We used the number of false positives for each individual leave-one-out experiment for a TF as the *rank* of the experiment. Therefore, a high rank indicated poor performance.

## Availability of supporting data

The data set, supplementary data, source codes (C#), and figures supporting the results of this article are available in http://biogrid.engr.uconn.edu/mlconsensus/.

## Appendix A: The naïve sorting-based multiple sequence alignment algorithm

The assumption behind this algorithm is that the core region is shared by all sites, and therefore on average, positions in short sites are more likely to constitute the core region (than positions in long sites). The steps of the algorithm are as follows: 

1. Sort all binding sites from shortest to longest.

2. Take the shortest site that is yet unaligned. If more than one sites have the smallest length, pick one in random. This makes up the initial alignment, A.

3. Compute C, the consensus, from A.

4. Let s be the next shortest, unaligned site.

5. If such an s does not exist, go to step 8. Otherwise,

6. Shift s along C from left to right. Find the alignment which produces highest score of t with respect to C. Add t to A at this alignment.

7. Go to step 3

8. Output: A is the complete multiple sequence alignment of S.

It can be observed that the order of choosing sites affects the resultant alignment, and a heterogeneous short site is likely to negatively impact the rest of the alignment. However, according to the assumption of the algorithm, if the short site contains the core region then it will not be heterogeneous.

## Appendix B: Construction of ROC curves

Let TP, TN, FP and FN denote the number of true positives, true negatives, false positives, and false negatives, respectively.

*False Positive Rate*, or FPR, is defined as the fraction of incorrectly classified known negative examples. Similarly, *True Positive Rate*, or TPR, is defined as the fraction of correctly classified known positive examples.

Let _*N*TF_ be the number of TFs for the given species. Let _TF*i*_ be the *i*-th TF. Let $N_{\mathrm{BS}}^{i}$ be the number of known binding sites for _TF*i*_. A leave-one-out cross-validation is conducted for each of the $N_{\mathrm{BS}}^{i}$ binding sites. If a known negative example scores more than the known positive example, it is considered as a false positive.

We computed an ROC (Receiver Operating Characteristic) curve for each configuration over each species. FPR and TPR were placed along x-axis and y-axis, respectively, and the curve indicates the TPR obtained at different values for FPR. The computation for each configuration was done in three steps. At first, we computed TPR and FPR for each leave-one-out experiment involving a known binding site. Next, these values were averaged over all BSs for each TF. Lastly, these values were further averaged over all TFs for a given species.

### 

#### 

##### Step One: Individual binding sites

Let _BS*j*,*i*_ be the *j*-th BS of _TF*i*_. Let _FPR*max*_be the maximum false positive rate considered for drawing the ROC curve. We used _FPR*max*_=0.20, or 20%. Let the range 0≤FPR≤_FPR*max*_ be divided into *M* equal slots. Let ${\mathrm{FPR}}_{k}^{\mathrm{slot}}$ denote the false positive rate corresponding to the *k*-th slot.

Let _FP*j*,*i*_be the number of false positives in the leave-one-out run which involves _BS*j*,*i*_ as the known positive binding site. Let _FPR*j*,*i*_be the observed false positive rate. For any given *allowable* false positive rate, if _FPR*j*,*i*_ is greater than the allowable FPR, the given configuration will not be able to identify the known positive example. _*Tj*_(*i*,*k*) denotes whether the known positive example could be identified (i.e., occurrence of a true positive) by setting the allowable FPR equal to the false positive rate for the *k*-th FPR slot.

(11) $$T_{j}(i,k)=\left\{\begin{array}{cc} 1 & :{\mathrm{FPR}}_{j,i}\leq {\mathrm{FPR}}_{k}^{\mathrm{slot}}\\ 0 & :\mathrm{otherwise} \end{array}\right)\;,$$

for $1\leq j\leq N_{\mathrm{BS}}^{i},\;1\leq i\leq N_{\mathrm{TF}},\;1\leq k\leq M$ .

##### Step Two: Averaging over all BSs for a given TF

For _TF*i*_, let _*T*BS_(*i*,*k*) be the average number of true positives obtained by setting the allowable FPR equal to the false positive rate for the *k*-th FPR slot.

(12) $$T_{\mathrm{BS}}(i,k)=\frac{1}{N_{\mathrm{BS}}^{i}}\cdot \overset{N_{\mathrm{BS}}^{i}}{\underset{j=1}{\sum }}T_{j}(i,k)$$

for 1≤*i*≤*N*_TF_, 1≤*k*≤*M*.

##### Step Three: Averaging over all TFs for a species

Let *T*_TF_(*k*) be the average number of true positives obtained by setting the allowable FPR equal to the false positive rate for the *k*-th FPR slot across all TFs.

(13) $$T_{\mathrm{TF}}(k)=\frac{1}{N_{\mathrm{TF}}}\cdot \overset{N_{\mathrm{TF}}}{\underset{i=1}{\sum }}T_{\mathrm{BS}}(i,k)$$

for 1≤*k*≤*M*. The ROC curve is produced by plotting *T*_TF_(*k*) at *k*-th FPR slot.

We considered only 0%–20% false positive rate for computing the area under an ROC curve. Since the FPR slots are discrete, we used the sum of TPR values in the mentioned FPR range as the area under an ROC curve.

## Competing interests

Financial competing interests

### 

· In the past five years have you received reimbursements, fees, funding, or salary from an organization that may in any way gain or lose financially from the publication of this manuscript, either now or in the future? Is such an organization financing this manuscript (including the article-processing charge)? If so, please specify. No

· In the past five years have you received reimbursements, fees, funding, or salary from an organization that may in any way gain or lose financially from the publication of this manuscript, either now or in the future? Is such an organization financing this manuscript (including the article-processing charge)? If so, please specify. No

· Do you hold any stocks or shares in an organization that may in any way gain or lose financially from the publication of this manuscript, either now or in the future? If so, please specify. No

· Do you hold or are you currently applying for any patents relating to the content of the manuscript? Have you received reimbursements, fees, funding, or salary from an organization that holds or has applied for patents relating to the content of the manuscript? If so, please specify. No

· Do you have any other financial competing interests? If so, please specify. No

Non-financial competing interests

### 

· Are there any non-financial competing interests (political, personal, religious, ideological, academic, intellectual, commercial or any other) to declare in relation to this manuscript? If so, please specify. No

## Author’s contributions

CHH planned and directed the research, described the model, and proposed the naïve sorting-based multiple sequence alignment algorithm. SQ implemented the model and methodology, carried out experiments, and made statistical evaluation of the outcome. Both authors read and approved the final manuscript.
